# Design Thinking as a Method for Developing Caregiver (and Other) Interventions

**DOI:** 10.1093/geroni/igab046.1530

**Published:** 2021-12-17

**Authors:** Leila Aflatoony, Molly Perkins, Drenna Waldrop, Kenneth Hepburn

**Affiliations:** 1 Georgia Tech, Atlanta, Georgia, United States; 2 Emory University of Medicine, Atlanta, Georgia, United States; 3 Emory University, Atlanta, Georgia, United States

## Abstract

“Design Thinking,” an innovative, human-centric approach to problem-solving, seeks to ensure that design efforts “solve the right problem.” This presentation describes the Design Thinking process and illustrates its use in the context of three design studio sessions with of family caregivers of patients at the Integrated Memory Care Clinic (IMCC), a comprehensive medical home for persons living with dementia. The Design Thinking process entails five steps – Empathize, Define, Ideate, Prototype, Test – that engage consumers/end-users to identify, as precisely as possible, the issues or concerns that are most important to them and to further identify the possible solutions that seem to most fully address these concerns. The process can be described as one of divergent and convergent thinking. In the first session, the Empathize phase, IMCC caregivers were asked to think as broadly as possible about needs not being met by IMCC. These topics were reviewed more convergently in the second session, the Define phase; here the participants agreed on a shorter, prioritized list of needs to be addressed. In the third session (that combined the Ideate and pre-Prototype stages), participants identified 14 topics (interventions) they felt should be included in this program. Finally, in the Test phase, they assessed the topics and agreed that the most important need IMCC could address would be to provide a comprehensive orientation program for new caregivers. IMCC clinicians concurred with the salience of the problem to be solved and saw addressing it as contributing substantially to the improvement of IMCC clinical care.

